# The Contribution of Human Antimicrobial Peptides to Fungi

**DOI:** 10.3390/ijms26062494

**Published:** 2025-03-11

**Authors:** Qiaoxi Zhang, Kitman Choi, Xiaoyue Wang, Liyan Xi, Sha Lu

**Affiliations:** Department of Dermatology and Venereology, Sun Yat-sen Memorial Hospital, Sun Yat-sen University, #107 Yanjiang West Rd., Guangzhou 510120, China; zhangqx28@mail2.sysu.edu.cn (Q.Z.); choikm@mail2.sysu.edu.cn (K.C.); wxy_1048686617@163.com (X.W.); xiliyan@mail.sysu.edu.cn (L.X.)

**Keywords:** fungi, human antimicrobial peptides, antifungal strategies, defensins, cathelicidins, *Candida*, *Aspergillus*

## Abstract

Various species of fungi can be detected in the environment and within the human body, many of which may become pathogenic under specific conditions, leading to various forms of fungal infections. Antimicrobial peptides (AMPs) are evolutionarily ancient components of the immune response that are quickly induced in response to infections with many pathogens in almost all tissues. There is a wide range of AMP classes in humans, many of which exhibit broad-spectrum antimicrobial function. This review provides a comprehensive overview of the mechanisms of action of AMPs, their distribution in the human body, and their antifungal activity against a range of both common and rare clinical fungal pathogens. It also discusses the current research status of promising novel antifungal strategies, highlighting the challenges that must be overcome in the development of these therapies. The hope is that antimicrobial peptides, as a class of antimicrobial agents, will soon progress through large-scale clinical trials and be implemented in clinical practice, offering new treatment options for patients suffering from infections.

## 1. Introduction

Fungi are delicate and ubiquitous organisms that play a crucial role in complex ecosystems. Fungal infections represent a significant public health concern for human health [1]. These infections can lead to two major types of diseases: superficial fungal diseases and invasive or systemic infections. The impact ranges from superficial infections that affect quality of life to systemic involvement that can be life-threatening. Recent global estimates indicate that approximately 6.5 million cases of invasive fungal infections occur each year, leading to 3.8 million deaths, with around 2.5 million (68%) of these deaths directly attributable to fungal infections [2,3,4]. Specifically, diseases such as invasive aspergillosis, *Candida* bloodstream infections, *Pneumocystis* pneumonia, and cryptococcal meningitis exhibit significant incidence and mortality rates. For example, the annual incidence of *Candida* bloodstream infections is approximately 1.56 million, with a mortality rate of 63.6% [4]. These fungal infections not only pose a major threat to global public health but also impose a heavy burden on healthcare resources and economic systems. The treatment of these infections often requires expensive antifungal medications and prolonged hospital stays, further exacerbating the socio-economic burden.

However, clinical practice relies on just three main classes of antifungal drugs—azoles, polyenes, and echinocandins—while additional agents like 5-flucytosine are used as adjuncts [5,6,7]. The limited range of these drugs, combined with their widespread use, has facilitated the emergence of drug-resistant strains of *Candida*, *Cryptococcus*, and *Aspergillus*. Moreover, in the past three decades, only one new class of antifungal agents has been introduced, leaving many common invasive fungal infections, including those caused by resistant strains, inadequately treated [8,9,10,11,12]. This clearly highlights that the development of antifungal drugs has not kept pace with the growing clinical demand. The high mortality rates associated with invasive fungal infections, the need for prolonged treatment courses, and the narrow spectrum of action and cross-resistance among existing antifungals all underscore the urgent need for innovation [13,14,15]. There is a critical demand for safer treatments with reduced toxicity, as well as novel antifungal agents with improved efficacy. Improving the accessibility of fungal infection treatment options and developing effective preventive measures are crucial strategies to alleviate the global burden and economic pressure associated with these infections.

AMPs are small proteins found in many organisms that target and inhibit the growth of microorganisms, including bacteria, fungi, and viruses [16,17,18]. As of January 2024, the Antimicrobial Peptide Database (APD) contains 3940 AMPs, with 2463 of them derived from animals [19]. This number is expected to continue rising. AMPs are a key part of the innate immune system and serve as the first line of defense against pathogens [20,21,22,23,24]. These peptides are naturally produced by a variety of organisms, including plants, animals, and microbes [25,26]. Because of their evolutionary conservation, many AMPs share similarities across different species, including mammals and humans. Numerous antimicrobial peptides from plants and other sources have been shown to exhibit strong antifungal properties. For example, *bCp12A*, an AMP derived from hydrolyzed casein in buffalo milk, demonstrates significant antimicrobial activity against *Staphylococcus aureus* and could be used to control bacterial contamination in milk, with potential applications in agriculture and food safety [27,28]. In a 2023 study, the antimicrobial peptide *CB-M* was found to have direct antifungal activity against gray mold and also helped induce resistance to gray mold in cherry tomato fruit [29]. Likewise, human AMPs are being progressively identified for their effectiveness against a broad spectrum of fungi. In this review, we have discussed the role of human AMPs in the treatment of fungal infections.

## 2. Human Antimicrobial Peptides

Human AMPs are found throughout the human body, including in organs like the skin, respiratory tract, and digestive system, as well as in immune cells and bodily fluids such as saliva, tears, and breast milk [30,31,32,33]. The classification of human AMPs currently lacks a standardized system. The most widely adopted approach categorizes AMPs based on their peptide family, including defensins, transferrins, and histones. Alternative classification schemes exist based on functional specificity (antibacterial, antifungal, or antiviral activity) or anatomical distribution (production sites or localization within the human body). The combination of the GEO transcriptome database with existing studies has provided new insights into the expressional levels and functions of human AMPs [34,35]. The distribution and concentration of these peptides often correspond to the types of local pathogens. For instance, Histatin 5 is present at much higher levels in human saliva than in sweat or on the skin, where it plays an important role in fighting bacteria that cause dental cavities and certain *Candida* species, helping prevent oral thrush in individuals with healthy immune systems [36,37].

For some AMPs that are constantly expressed, their levels can change based on other factors. For example, the expression of defensins in the endometrium varies with different stages of the menstrual cycle and in response to microbial infections [38,39,40]. These peptides are a key part of the body’s natural defense system, with broad-spectrum antimicrobial activity against bacteria, fungi, viruses, and even some cancer cells [41,42,43,44,45]. In addition, the antimicrobial peptide database includes peptides that aid in wound healing, reduce inflammation, and have potential anti-diabetic effects.

In summary, AMPs are becoming increasingly recognized for their diverse roles in immune defense, thanks to their widespread presence and evolutionarily conserved structures. Many human AMPs also show antifungal activity, making them promising candidates for developing new antibiotics and other treatments.

## 3. Antifungal Activities of Human AMPs

A wide range of AMPs have been identified so far. In humans, the main families of AMPs include defensins, cathelicidins, transferrin (LF), and histones (Hst). Lysozyme, the first human AMP to be discovered, was found in nasal secretions [46,47]. While lysozyme mainly targets the bacterial cell wall component peptidoglycan, its activity against fungi and viruses is relatively weak. LL-37, the only known human member of the cathelicidins family, exhibits antifungal properties against several common fungal pathogens, such as *Candida* and *Aspergillus*.

These peptides show varying levels of activity, and Table 1 summarizes the human AMPs currently known and their antifungal properties. Research on the antifungal effects of AMPs has primarily focused on their in vitro antimicrobial activity, stability, hemolytic toxicity, and efficacy in animal models. With the advancement of peptide engineering, many researchers have modified natural AMPs through truncation or chemical modification, or synthesized entirely new peptides using amino acid-based chemical synthesis. Synthetic peptide design based on natural AMP templates focuses on two strategic goals: first, the identification and enhancement of bioactive motifs essential for antimicrobial function; second, the systematic removal or modification of sequence regions contributing to cytotoxicity and proteolytic instability [48]. This dual-focused strategy facilitates the creation of next-generation antimicrobial agents with optimized therapeutic potential. For instance, rational modification of the PvD1 γ-core sequence, including charge optimization and length reduction, yielded the γ33-41 PvD1 ++ peptide. This nine-amino acid derivative showed enhanced antifungal activity and represents a promising candidate for developing cost-effective antifungal therapeutics [49,50].

Despite the growing interest in human AMPs, their stability in vivo remains a relatively underexplored aspect. Although a significant number of AMPs have been discovered and characterized, many remain unidentified, highlighting the ongoing significance of human peptidome research for future scientific advancements [51]. In the following sections, this review will examine the antifungal effects of AMPs on fungi responsible for superficial infections, as well as those causing systemic infections, with a focus on recent research advancements in these areas.

**Table 1:** Human AMPs currently known and their antifungal properties.

Antimicrobial Peptide	Source (Human)	Antifungal Activity	Reference
Lactoferrin (LF)	Mucosal secretions, saliva, milk	*Candida albicans*, *Candida kefyr*, *Candida krusei*, *Candida parapsilosis*, *Cryptococcus neoformans*, *Aspergillus fumigatus*	[52,53,54,55,56]
Histatin-5 (Hst 5)	Saliva	*Candida albicans*, *Candida kefyr*, *Candida, Candida parapsilosis*, *Cryptococcus neoformans*, *fumigatus*	[57,58,59,60,61,62,63,64,65]
Histatin-1 (Hst 1)	Oral cavity	*Candida albicans*, *Candida krusei*	[66]
(Hst 2)	Oral cavity	*Candida albicans*, *Candida krusei*	[66,67]
Ribonuclease A (RNase A)	Vertebrates, chromosome 14	*Candida albicans*, *Candida tropicalis*	[68,69,70]
RNase 1	Pancreas	*Candida albicans*, *Candida tropicalis*	[69,71]
RNase 2	Eosinophils	*Candida albicans*, *Candida tropicalis*	[70]
RNase 5 (Angiogenin)	Neurotoxin	*Candida albicans*, *Candida tropicalis*	[72]
RNase 7	Skin	*Candida albicans*, *Candida tropicalis*, *Aspergillus fumigatus*	[73,74,75]
Dermcidin (DCD)	Sweat glands	*Staphylococcus aureus*, *Staphylococcus epidermidis*, *Escherichia coli*, *Enterococcus faecalis*, *Candida albicans*	[76,77,78]
LL-37	Airways, oral cavity, tongue, esophagus, epididymis, small intestine	*Candida albicans*, *Candida kefyr*, *Candida krusei*, *Candida parapsilosis*, *Cryptococcus neoformans*, *fumigatus*, *Trichophyton rubrum*, *Microsporum canis*	[79,80,81,82,83,84,85,86,87]
Hepcidin	Liver	*Candida albicans*, *Candida tropicalis*, *Aspergillus fumigatus*	[88,89,90,91,92]
Vasostatin-1	Endocrine, neuroendocrine, and neuronal cells	*Candida albicans*, *Candida tropicalis*, *Candida kefyr*, *Aspergillus fumigatus*, *Fusarium solani*, *Sporothrix schenckii*	[93]
Alpha-defensins (HD5, HD6)	Intestinal Paneth cells, neutrophils	*Candida albicans*, *Aspergillus fumigatus*, *Candida glabrata*, *Cryptococcus neoformans*	[94,95,96,97]
Beta-defensins (hBD-1, hBD-2, hBD-3)	Epithelial cells (skin, respiratory tract, urogenital tract)	*Candida albicans*, *Candida glabrata*, *Aspergillus fumigatus*, *Cryptococcus neoformans*, *Trichophyton rubrum*	[98,99,100,101,102,103,104,105,106,107,108,109,110]

### 3.1. Malassezia

*Malassezia* is a lipophilic yeast that is commonly found on the surface of human skin [111,112]. A study using high-throughput sequencing to investigate the microbiomes of the forehead, scalp, and arms of healthy individuals found that the relative abundance of *Malassezia* species was significantly higher than that of other fungal taxa [113,114]. Overgrowth of *Malassezia* can lead to conditions such as tinea versicolor and *Malassezia* folliculitis and is also closely associated with skin disorders like seborrheic dermatitis and atopic dermatitis. A case-control study revealed that several human defensins (e.g., β-defensin 2, β-defensin 3, LL-37, S100A7, and RNase 7) were significantly elevated in patients with tinea versicolor, suggesting that the expression of these AMPs is linked to *Malassezia* infection [115]. Two forms of the cathelicidin peptide, LL-37 in human neutrophils and CRAMP in murine neutrophils, have been shown to inhibit the growth of *Malassezia* species, with minimum inhibitory concentrations (MICs) ranging from 20 to 30 μM [115,116]. Additionally, another study demonstrated that a peptide analogous to frog skin secretion, P5, and a peptide derived from recombinant antibody constant regions exhibited effective antimicrobial activity against *Malassezia furfur*.

However, it has also been observed that in patients with eczema, the secretion of LL-37 correlates with the severity of inflammation triggered by *Malassezia* infection. This suggests that the immunomodulatory effects induced by LL-37 may also play a role in the pathogenesis of eczema. As a result, further research is needed to better understand the interactions between fungal pathogens and AMPs, particularly in the context of skin conditions. Additionally, the development of new synthetic AMPs should take into account their potential immunomodulatory effects. Achieving a balance between antimicrobial activity and the modulation of inflammatory responses in both in vivo and in vitro studies is essential for advancing the clinical application of AMPs.

### 3.2. Trichophyton

*Trichophyton* species are important pathogens responsible for superficial skin infections and/or infections of its appendages, such as tinea corporis, tinea pedis, onychomycosis, and others [117,118]. Infected areas typically present with symptoms such as pruritus and desquamation [118,119]. The most commonly isolated species in clinical settings include *Trichophyton rubrum*, along with other species like *T. mentagrophytes*, *Microsporum canis*, and *Epidermophyton floccosum* [120]. Studies [116,121] have shown that the expression of cathelicidin peptides is upregulated in skin biopsies from patients diagnosed with tinea pedis and tinea corporis. In vitro experiments have also demonstrated that exposure to *T. rubrum* induced an increase in the expression of mRNA encoding AMPs in keratinocytes [116].

While most dermatophyte infections are not directly life-threatening, the concern worldwide about the increasing resistance of dermatophytes to standard antifungal treatments is growing [122,123,124,125]. *Trichophyton indotineae*, a strain within the *T. mentagrophytes*/*T. interdigitale* complex, has recently emerged as a drug-resistant pathogen [125,126]. On the other hand, the lengthy treatment courses required for onychomycosis often lead to poor patient adherence, reducing treatment effectiveness and lowering cure rates.

In this context, AMPs offer a promising alternative, potentially overcoming the limitations of current therapies [2,59]. Over the past two decades, research on AMPs targeting dermatophytes has intensified. One such AMP, the cathelicidin-like peptide Pa-MAP [121], which is rich in alanine analogs, exhibits effective antifungal activity against both *T. mentagrophytes* and *T. rubrum*. Similarly, a lactoferrin-derived peptide, Compound 5, has demonstrated superior antifungal effects in human onychomycosis models, outperforming commonly used antifungal agents like terbinafine and amorolfine [127,128,129]. Currently, the cyclic heptapeptide Novexatin is undergoing clinical trials for the treatment of onychomycosis [130,131]. Undoubtedly, human AMPs such as LL-37, human β-defensins, and RNase 7 hold significant potential as therapeutic agents for skin fungal infections [132,133,134,135,136,137,138].

In addition to the aforementioned human-derived AMPs, natural cyclic heptapeptides isolated from the marine bacterium *Stylissa caribica* and the synthetic cyclopolypeptide 8 have also shown antifungal activity against dermatophytes like *T. mentagrophytes* and *T. rubrum* (MIC 6 µg/mL) [139,140,141]. A cyclic peptide, Phaeofungin, derived from algae, has demonstrated antifungal activity against *T. mentagrophytes* (MIC 4 µg/mL). These findings suggest that cyclic structures may play a role in combating superficial infections caused by dermatophytes such as tinea pedis and tinea unguium. A recent genomic analysis of skin fungal pathogens (*Trichophyton*, *Microsporum*) revealed an enrichment of LysM-containing domains, which could serve as binding regions for cyclic heptapeptides [140,142,143,144]. Notably, a limitation of current research is lacking the studies investigating the specific binding sites and conformations of AMPs in *Trichophyton* species, which may hinder accurate predictions of how structural modifications affect antimicrobial efficacy.

### 3.3. Candida

*Candida* species are integral to the human microbiome, playing a key role in the structure and metabolic functions of microbial communities. As we know, *Candida* species can colonize superficial areas such as the oral mucosa, conjunctiva, and skin, leading to conditions like intertrigo and oral thrush [145,146,147,148,149]. *Candida* can be also detected in gastric and fecal samples, suggesting its presence in the gastrointestinal tract, although it remains unclear whether this represents transient colonization or long-term persistence.

In the past two decades, *Candida* infections have emerged as a major public health issue [150,151]. In the United States, *Candida* is the fourth leading cause of hospital-acquired bloodstream infections, with a mortality rate as high as 40% [152]. More seriously, *Candida* can invade the bloodstream, causing systemic infections across various organs [153,154]. These severe infections predominantly occur in immunocompromised patients, such as those on long-term immunosuppressive therapy or with HIV [155,156,157]. In addition, due to the limited range of antifungal drugs available for clinical use, there has been a decline in the sensitivity of *C. albicans* to azoles, and *C. auris* has shown multidrug resistance to various antifungal agents [158,159].

As a result, researchers are actively seeking new therapeutic strategies to combat *Candida* infections. AMPs are among the first molecules released by mucosal surfaces and are integral components of the innate immune response against fungal infections. Extensive research has shown that AMPs possess potent antifungal activity against *Candida* species, and their mechanisms of action are well understood. The ADP3 database alone records 910 AMPs with antifungal activity against *Candida* species. In 1994, dermaseptin I, extracted from frog skin, was the first AMP identified to exhibit antifungal activity against *C. albicans* in vitro [160,161]. AMPs with anti-Candida properties are widely distributed across diverse biological taxa. Representative examples include NaD1 from Nicotiana alata flowers and Gomesin from Acanthoscurria gomesiana [162,163]. Although the current repertoire of human-derived anti-Candida peptides is less extensive compared to those from insects and arachnids, significant progress has been made in characterizing human AMPs such as Psoriasin and β-defensin [164,165]. In addition, recent studies have progressively revealed the therapeutic value of modified human AMP derivatives in combating *Candida* infections. The human AMP family exhibits an intriguing pattern of structural homology with subtle variations among its members [166,167]. These naturally occurring variations offer a promising foundation for developing enhanced peptide-based therapeutics through structural modification approaches.

Subsequent studies have focused on the in vitro and in vivo antifungal activities of various AMPs, including human defensins (e.g., HBD-6, LL-37), lysozyme, and histone family peptides, all of which have shown effective antifungal properties against *Candida* species [30,168,169,170,171,172,173,174,175]. In experimental protocols, researchers commonly employ azole antifungal agents and melittin as positive controls [176]. The most representative MIC values are typically selected for subsequent mechanistic investigations and structural optimization studies. Due to the diverse range of diseases caused by *Candida*, in vivo models have been extensively developed to study the efficacy of AMPs. These include local skin infection models, corneal infection models, and systemic infection models in mice, which evaluate the impact of specific AMPs on overall survival and organ-specific fungal burden [177,178,179,180]. These critical animal experiments are indispensable for validating both the safety parameters and pharmacological efficacy of AMPs in clinical applications, necessitating strict protocol compliance to maximize therapeutic potential [181].

In addition to the well-studied AMPs, researchers have been exploring other potential antimicrobial peptides derived from human tissues. For example, a peptide fragment (2–2) of glyceraldehyde-3-phosphate dehydrogenase (GAPDH), isolated from human placenta, was found to enter *Candida* cells and induce apoptosis [182]. In 2023, a peptide, YY (PYY (1–6)), was discovered in human intestinal epithelial Paneth cells, offering a new approach for the packaging and delivery of antimicrobial peptides [183]. Furthermore, α-melanocyte-stimulating hormone (α-MSH), secreted by the skin, has been shown to reduce the viability of *C. albicans* and inhibit its hyphal formation [184,185,186].

However, these newly discovered peptides were not initially identified for their antifungal activity and may have other physiological roles. For instance, GAPDH is an essential enzyme in the glycolytic pathway, and α-MSH plays a regulatory role in various hormones, including growth factors, insulin, and aldosterone [182,186,187,188,189]. S100A7, an AMP secreted by human keratinocytes, demonstrates limited direct fungicidal activity against *Candida* species, but it exhibits significant antibiofilm properties [190]. This protein facilitates the disruption of fungal biofilms, thereby enhancing the penetration and efficacy of conventional antifungal agents [191]. These characteristics position S100A7 as a promising candidate for future combination therapies in the treatment of *Candida* infections. To develop these peptides as potential antifungal agents, it is crucial to balance their antifungal efficacy with their other biological activities to minimize potential side effects and adverse reactions.

### 3.4. Aspergillus

*Aspergillus* infections are the second most common fungal infections that lead to hospitalization, following those by *Candida* species [192,193,194,195]. The most common mode of infection is the inhalation of conidia from the air. Epidemiological data have identified several high-risk populations, particularly individuals with pre-existing pulmonary conditions such as chronic obstructive pulmonary disease (COPD), asthma, or cystic fibrosis, as well as those recovering from severe respiratory viral infections, including influenza and COVID-19 [193,196,197].

Among pathogenic *Aspergillus* species, *A. fumigatus* is the most prevalent in the environment and the most commonly implicated in clinical aspergillosis, followed by *A. niger* [198,199,200,201]. Studies have shown that in patients with chronic sinusitis complicated by *A. fumigatus* infection, the expression levels of LL-37 are upregulated. An in vitro study demonstrated that LL-37 inhibits the hyphal growth of *A. fumigatus* in a dose-dependent manner after 12 h of incubation [81]. In addition to LL-37, other common human antimicrobial peptides, such as human β-defensin (hBD) and lactoferrin, have also shown effective antifungal activity against *A. fumigatus* [56,202,203,204]. The antimicrobial efficacy of AMPs against *Aspergillus* species is influenced by multiple factors, including peptide concentration, fungal strain characteristics, the growth phase of the organism, and environmental conditions. These variables collectively determine the outcome of AMP-*Aspergillus* interactions and subsequent antifungal activity. On another hand, the host immune response to *Aspergillus* infection is significantly modulated by AMPs, which activate key immune effector cells such as neutrophils, macrophages, and dendritic cells. This activation enhances fungal pattern recognition and promotes phagocytic elimination of *Aspergillus* conidia and hyphae, thereby strengthening the host’s antifungal defense mechanisms [205].

Future research is needed for developing more diverse in vivo models to validate the in vivo efficacy and potential clinical applications of human antimicrobial peptides against *Aspergillus* species.

### 3.5. Cryptococcus

Like other fungi, many antimicrobial peptides have been shown to exhibit activity against *Cryptococcus* in vitro [206,207,208]. For example, a proline-rich peptide (2733 Da) isolated from a pig parotid gland extract demonstrated antifungal activity against clinical isolates of *Cryptococcus neoformans*, with an EC50 value of 2.2 μM [209].

However, unlike other fungi, *Cryptococcus* is a common cause of meningitis in clinical settings, often leading to a range of neurological and psychiatric symptoms in affected patients [208,210,211,212]. Currently, amphotericin B (AmB) is the most commonly used treatment for fungal-induced central nervous system infections [208,210]. Global guidelines suggest that both AmB deoxycholate (AmB–) and lipid formulations of AmB can be used as adjuncts to intravenous therapy. This highlights the critical role of lipophilicity in drug design, as it facilitates the drug’s passage across the blood–brain barrier to reach the therapeutic site.

Given the generally hydrophilic nature of antimicrobial peptides and the lipophilic characteristics of the blood–brain barrier, a 2010 study proposed several strategies to enhance the delivery of AmB to the brain, such as increasing the dosage, intrathecal injection, and using brain-targeted peptide-modified liposomes. In vitro experiments demonstrated that a self-assembled, cationic nanoparticle composed of a lipophilic cholesterol conjugate with a TAT peptide sequence (G3R6TAT) exhibited good antifungal activity against *Cryptococcus* growth, slightly surpassing that of AmB [213,214]. Additionally, in a meningitis rabbit model, this nanoparticle improved survival rates [214]. These findings provide evidence that this nanoparticle formulation effectively inhibits *Cryptococcus* growth and inflammatory responses in both cerebrospinal fluid and brain parenchyma.

### 3.6. Histoplasma capsulatum

*Histoplasma capsulatum* is typically found in the feces of birds and bats, and humans usually contract histoplasmosis through airborne inhalation of its spores. As a result, the primary site of fungal infection is commonly the lungs [215,216,217]. However, in immunocompromised individuals, such as patients with HIV/AIDS, the infection can disseminate through the bloodstream to other organs, including the skin, bone marrow, brain, liver, spleen, and lymphatic system, leading to progressive disseminated histoplasmosis.

In in vitro studies, mouse macrophages that produce human defensins have been shown to effectively inhibit the replication of *H. capsulatum* within cells, thus controlling the spread of the infection [218]. Additionally, a member of the human heat shock protein 60 (HSP60) family, HIS-62, has been found to offer protective effects against pulmonary histoplasmosis in mice [219].

Furthermore, some newly synthesized antimicrobial peptides, such as Lys-Nva-FMDP, have demonstrated the ability to inhibit the growth of *H. capsulatum* in its yeast form, as well as exhibit promising in vivo antifungal activity in organ load tests in mice [220,221]. These novel synthetic peptides, with clearly defined structures, were designed based on the structural and functional studies of natural antimicrobial peptides [221,222,223,224]. Due to their purpose-driven synthesis, the antifungal mechanisms of these artificially synthesized peptides are more clearly understood.

### 3.7. Paracoccidioides brasiliensis

*Paracoccidioides brasiliensis* is a dimorphic fungus capable of causing disease in both healthy and immunocompromised hosts [225]. The risk of developing disseminated paracoccidioidomycosis is particularly high in immunocompromised individuals. Research on peptides involved in *P. brasiliensis* infection in humans has largely focused on the infection process itself [226,227,228,229]. WI-1 is a surface protein found on *P. brasiliensis* cells that has been shown to significantly induce inflammatory responses in human paracoccidioidomycosis, serving as a target antigen for cell-mediated immunity in this disease [230,231,232]. Such target antigens, by synthesizing similar structures that preserve their antigenicity while reducing their toxicity, present an ideal candidate for antimicrobial peptide-based vaccines.

### 3.8. Mucorales

Several fungi from the order *Mucorales* can cause mucormycosis in humans, a severe infection that can affect multiple organ systems [233,234,235,236,237,238]. These fungi include species from the genera *Mucor*, *Rhizopus*, or *Rhizomucor species* [239,240,241]. The most common sites of infection are the nasal and cerebral regions, as well as the lungs [242]. Mucormycosis is most commonly observed in immunocompromised patients, individuals with poorly controlled diabetes (especially those with diabetic ketoacidosis), and patients receiving iron chelation therapy with deferoxamine [242,243,244]. Among these, *Rhizopus* is the most frequent causative agent of mucormycosis. Mortality rates are higher in patients with prolonged neutropenia, disseminated disease, or brain infections [245].

Research on *Rhizopus* primarily focuses on the structure, active sites, and key amino acids of enzymes such as *Rhizopus* aspartic proteinases, lipases, and glucosidases, which play significant roles in fungal virulence [246,247,248,249,250,251,252,253,254]. However, there is limited research on the hemolytic toxicity and cell-killing effects of *Rhizopus*, and no animal studies have been conducted to explore the potential of reducing fungal loads in *Rhizopus* infections [254,255,256,257,258]. This gap in research may be attributed to the challenges in constructing appropriate animal models for *Rhizopus* infection.

Recent studies, however, have identified newly synthesized peptides with effective antifungal activity against *Mucorales*. One such peptide inhibitor binds to the aspartic proteinase of *Rhizopus*, impacting its catalytic activity and thereby exerting antifungal effects [259]. This direction of research, focused on protein–protein interactions, is somewhat distinct from other fungal studies but shows promise [259,260]. Additionally, a study synthesized an antimicrobial undecapeptide with spore-killing activity, demonstrating its potential to control apple rot [261]. This progress highlights the faster pace of development in agricultural and food-related applications of antifungal peptides compared to that of clinical medical research [262,263].

### 3.9. Talaromyces marneffei

*T. marneffei* is one of the most important pathogenic thermally dimorphic fungi in China and Southeast Asia [264,265,266,267,268]. The prevalence of HIV/AIDS, particularly in China and other Southeast Asian countries, has led to *T. marneffei* infections becoming a significant opportunistic infection in AIDS patients [265,269,270,271]. Research on AMPs targeting *T. marneffei* is limited. The MP1 gene of *T. marneffei* appears to share similarities with the AFMP1 gene expressed by *Aspergillus fumigatus*. Therefore, engineering peptides that target AFMP1 may have potential therapeutic effects in controlling *T. marneffei* infections [272,273].

## 4. Mechanism by Human AMPs Activity

AMPs are considered promising broad-spectrum antimicrobial agents due to their lack of drug resistance, and their mechanisms of action have been extensively studied [274,275]. Unlike traditional antibiotics, AMPs possess unique structural features that enable them to interact electrostatically with fungal cell membranes, reducing the likelihood of resistance development [276,277,278]. While the exact antifungal mechanisms of AMPs are not fully understood, most of these peptides target fungal cell membranes. Several factors, including charge (cationic or anionic), size, amino acid sequence, conformation, hydrophobicity, and amphipathicity, can influence the activity and mechanism of AMPs. For instance, structural modification of HBD-1 through disulfide bond reduction enables the peptide to acquire antimicrobial activity against *C. albicans* [279].

In contrast to conventional antifungal agents that typically target single cellular components, AMPs primarily exert their effects through two main mechanisms: direct killing and immune modulation [280,281]. The direct killing mechanism can be further divided into membrane-targeted and non-membrane-targeted actions. Membrane-targeting peptides, such as defensins and LL-37, disrupt the membrane integrity by forming transient pores [282,283,284,285]. These membrane-targeting mechanisms can be explained by various models, including the barrel-stave model, carpet model, pore-forming model, and detergent model [286,287,288,289]. The oligomerization of peptides leads to alterations in the fungal cell membrane, causing leakage of cellular contents and ultimately leading to cell death [290]. Using LL-37 as an illustrative example, this multifunctional antimicrobial peptide induces *C. albicans* apoptosis through multiple mechanisms. As a pore-forming peptide, it creates membrane pores at nanomolar concentrations, leading to ion efflux and membrane destabilization [291,292]. Following cellular internalization, LL-37 interacts with intracellular targets while simultaneously stimulating endogenous ROS production, which contributes to mitochondrial membrane potential dissipation, a hallmark of apoptotic initiation [293]. The subsequent release of Ca2+ from the endoplasmic reticulum and its mitochondrial translocation further exacerbates organelle dysfunction [294]. Ultimately, cytochrome c release from mitochondria into the cytosol activates caspase proteases, executing the apoptotic cascade. Non-membrane-targeting peptides, such as pleurocidin, pyrrhocidin, and mersacidin, penetrate the cell membrane without directly disrupting it. Chromogranin A (CGA)-N46 exerts its antifungal activity by damaging the mitochondria, inducing vacuolization inside of the yeast cells, disturbing the nuclear envelope and inhibiting DNA synthesis by preventing DNA polymerase action. Instead, they interfere with critical intracellular processes, ultimately resulting in cell death [295,296,297,298].

In addition to their direct antimicrobial activity, AMPs also play a key role in immune modulation by regulating various signaling pathways [299,300,301,302]. They can recruit effector cells, such as phagocytes, to enhance both intracellular and extracellular killing. AMPs also promote macrophage differentiation and dendritic cell maturation through activation of immune cells, stimulation of immune cell proliferation, and the promotion of immunoglobulin and cytokine secretion such as tumor necrosis factor-α (TNF-α), interleukin-1 (IL-1), and IL-6. Specifically, LL-37 upregulates the expression of aryl hydrocarbon receptor (AHR) and retinoic acid-related orphan receptor γt (RORγt), while simultaneously enhancing the phosphorylation of Smad 2/3 and signal transducer and activator of transcription 3 (STAT3) proteins. These molecular events collectively drive the differentiation of native T cells toward the Th17 phenotype, which plays crucial roles in mucosal immunity and pathogen clearance [303]. Additionally, AMPs also exhibit additional biological functions for the regulation of wound healing processes. Those multifunctional capabilities contribute to tissue homeostasis and cellular turnover, highlighting the diverse physiological roles of AMPs in maintaining organismal integrity [304,305,306].

The diverse mechanisms of AMPs naturally lead to the hypothesis that combining peptides with complementary modes of action may yield synergistic effects. Given the evolutionary conservation of AMPs across species, researchers have focused on exploring combinations of human-derived and non-human AMPs. A notable example is the synergistic interaction between human LL-37 and plant-derived NaD1, which collectively enhance hyphal permeabilization and accelerate fungal cell death [307]. This strategy of leveraging complementary advantages from human and non-human peptides may unlock novel therapeutic options for combating multidrug-resistant fungal infections.

The therapeutic potential of human antimicrobial peptides for fungal infections shares conceptual similarities with vaccine development. Current clinical trial data indicate that topical AMP applications are generally well-tolerated, with few reported adverse events [308]. In preclinical studies examining systemic administration, AMPs have shown manageable cytotoxicity profiles in animal models. Engineered AMP variants and peptides from non-human sources, when designed with precise target specificity, typically demonstrate minimal effects on human cells [180]. Nevertheless, researchers must consider that as natural components of innate immunity, AMPs possess biological activities that extend beyond antifungal action, potentially including mechanisms that could affect normal cellular functions.

## 5. Sources and Modification of New Antifungal Agents

AMPs can be derived from animals, plants, and microorganisms, or they can be chemically synthesized [26,309,310,311,312]. AMP discovery has evolved through two distinct methodological paradigms. The traditional approach, grounded in the empirical screening of biological peptide libraries, systematically evaluates antimicrobial potential through iterative experimentation. Modern computational strategies, enabled by advances in bioinformatics, now allow for predictive identification of antimicrobial sequences directly from proteomic and genomic datasets. The primary methods for obtaining AMPs include direct isolation from natural sources, heterologous expression, and chemical synthesis. Direct isolation from natural sources involves a combination of solvent extraction, chromatography, and analytical techniques. Chemical synthesis, including solid-phase synthesis, is also commonly employed [313,314]. Among these, chemical synthesis and recombinant production provide reliable, cost-effective means of producing AMPs with high efficiency.

Dissatisfied with the existing AMPs, researchers continue to identify novel AMPs. In terms of direct isolation, advances in sequencing technologies and AI-driven deep learning systems are increasingly used to predict and isolate potential AMPs from biological sources [34,315,316]. For example, one innovative study utilized multiple natural language processing models to successfully identify AMPs with antimicrobial potential from the human gut microbiome [317].

In synthetic AMP development, some synthesized peptide analogs exhibit broader activity against target organisms compared to their natural counterparts. As a result, peptide engineering has emerged as a key area of focus, involving the molecular design and modification of peptides to improve their antimicrobial activity or reduce toxicity. Techniques such as the synthesis of dendrimeric peptides and the cyclization of polypeptides have shown promise in enhancing antifungal activity [318,319,320]. Additionally, positively charged peptides rich in lysine have been associated with reduced toxicity and increased antifungal effectiveness, and could even been used to create sites that bind to nanoparticles to facilitate their delivery [321,322,323,324,325,326,327]. This paradigm shift introduces new scientific considerations, particularly in source organism selection. Microbial communities have emerged as particularly promising discovery platforms, offering three distinct advantages: (1) unparalleled genetic diversity within their collective genomes, (2) evolutionary adaptations shaped by persistent host–microbe interactions, and (3) competitive pressures driving antimicrobial innovation [328,329,330,331,332]. These inherent biological features position microbial ecosystems as rich, largely untapped reservoirs of AMPs with significant translational potential. Overall, peptide engineering relies on a precise understanding of peptide primary and secondary structures, a process that is closely linked to computational methods [333,334]. We anticipate that future advancements in peptide modification strategies will provide more effective options for novel antifungal drugs.

## 6. Future Directions

Current research on AMPs focuses primarily on their effects on pathogens and their potential applications. Studies are generally divided into in vitro experiments, in vivo experiments, and mechanistic investigations [335]. Regarding fungi, in vitro research typically includes determining the MIC of AMPs against various clinical isolates to evaluate their antimicrobial activity. Additionally, AMPs’ stability under extreme conditions, such as high salinity, extreme pH, or in the presence of proteases, is tested to determine their potential in vivo applicability. Mechanistic studies are also mainly conducted in vitro, often focusing on comparing the expression of various inflammatory factors to understand the immune-modulatory effects of AMPs and their involvement in downstream signaling pathways. Researchers also use techniques such as electron microscopy to visually capture changes in fungal cells after AMP treatment. As previously mentioned, most human AMPs (e.g., HBD, LL-37) induce fungal cell death through membrane interactions, leading to membrane disruption and nuclear envelope dissolution, phenomena observable under electron microscopy. However, these methods are no longer novel. Currently, there is a greater interest in directly confirming molecular interactions after membrane binding, rather than relying on models to hypothesize these interactions.

In vivo studies are less common and mainly focus on *Candida* species. This is likely due to the diverse infection sites of *Candida* (e.g., corneal, vaginal, skin, and systemic infections), with superficial localized infection models being much easier to establish than systemic infection models [336,337,338]. Unfortunately, many fungi that cause fatal infections, such as *Microsporum* and *Mucor*, have not been studied with regard to the establishment of infection models. It is clear that there is still a long way to go in discovering novel clinical treatments for these fungi. Large-scale clinical trials are also essential for the approval of new drugs. Unlike infections such as influenza or COVID-19, systemic fungal infections are relatively rare, making such trials both costly and difficult to conduct [339]. A promising approach is to conduct small-scale studies initially, followed by the development of an international platform for adaptive clinical trial design, where data can be aggregated and analyzed through meta-analysis.

## 7. Conclusions

AMPs represent a fascinating and diverse class of molecules with vast potential for treating infections, particularly in an era where antimicrobial resistance is becoming an increasingly urgent problem. These peptides are naturally occurring across various organisms. They have evolved to perform multiple functions, from directly killing pathogens to modulating immune responses. One of the most compelling advantages of AMPs is their effectiveness against resistant fungi, including species of *Candida*, *Aspergillus*, and *Talaromyces*, which have become significant threats, especially in immunocompromised populations. This broad-spectrum antifungal activity, coupled with their rapid action, makes AMPs promising candidates in the fight against infections that are increasingly difficult to treat with conventional antifungal drugs. However, while their potential is immense, there are significant hurdles to overcome before AMPs can be widely used in clinical settings.

First, their stability in vivo remains a major challenge. AMPs are often rapidly degraded in the body, limiting their therapeutic potential. Additionally, their high cost of production and potential toxicity, especially at higher concentrations, require careful optimization. As with many promising therapeutic candidates, the clinical use of AMPs hinges on balancing their antimicrobial potency with safety and patient tolerance.

Furthermore, while we have a growing understanding of the mechanisms through which AMPs combat pathogens, there is still much to learn about their precise interactions with different microorganisms. For instance, the role of AMPs in modulating the immune system, particularly in the context of inflammatory diseases or fungal infections, remains underexplored. Given the complexity of the immune response, AMPs’ dual roles—both as antimicrobial agents and immune modulators—need further investigation to ensure their efficacy and safety in long-term treatments.

In conclusion, while AMPs hold great promise, their path to clinical use will require ongoing research. With continued advancements in peptide engineering and deeper insights into their mechanisms of action, AMPs may one day become a cornerstone of the treatment for fungal infections and other microbial diseases, offering a much-needed alternative to current therapies.
